# Age-Related Alterations in Endothelial Function of Femoral Artery in Young SHR and WKY Rats

**DOI:** 10.1155/2014/658479

**Published:** 2014-03-20

**Authors:** Angelika Puzserova, Veronika Ilovska, Peter Balis, Peter Slezak, Iveta Bernatova

**Affiliations:** ^1^Institute of Normal and Pathological Physiology, Centre of Excellence for Examination of Regulatory Role of Nitric Oxide in Civilization Diseases, Slovak Academy of Sciences, Sienkiewiczova 1, 813 71 Bratislava, Slovakia; ^2^Institute of Medical Biochemistry, Jessenius Faculty of Medicine, Comenius University, Mala Hora 4, 036 01 Martin, Slovakia

## Abstract

The present study was designed to evaluate the effects of vascular aging in juvenescence on endothelial function in femoral arteries and to assess differences between normotensive and hypertensive rats. The aim of the study was to determine if age affected nitric oxide- (NO-) mediated relaxations in normotensive and hypertensive rats. Juvenile (7-week-old) and young adult (22-week-old) male Wistar-Kyoto (WKY) and spontaneously hypertensive rats (SHR) were used in this study. Femoral artery (FA) reactivity was determined by wire myograph and NO synthase activity by conversion of [^3^H]-L-arginine. During juvenescence systolic blood pressure (tail-cuff) increased significantly only in SHR, while NO synthesis decreased significantly in both strains. Endothelium-dependent relaxations to acetylcholine were reduced in the FA of SHR compared to age-matched WKY at both ages, yet these parameters were unchanged in adult rats compared with juvenile animals. The NO-dependent component of vasorelaxation was markedly reduced, whereas the NO-independent component was increased in adult compared to juvenile rats in both strains. The endothelial dysfunction in SHR at both ages was associated with reduction of NO-independent mechanisms. In conclusion, aging in early periods of life was associated with reduction of vascular NO production and bioavailability in both strains investigated. This reduction was however fully compensated by accentuation of NO-independent mechanisms.

## 1. Introduction

Studies using experimental animal models as well as clinical research have indicated that hypertension is associated with endothelial dysfunction, a state in which the endothelial disorder leads to reduced vasodilation and increased vasoconstriction. Despite enormous research effort, the causal relationship between endothelial dysfunction and hypertension has remained unclear.

Endothelial dysfunction develops due to various risk factors, including aging of the organism. The latter, however, may be the causative agent even in the absence of established risk factors [1, 2]. Moreover, aging and hypertension have been identified as major risk factors for cardiovascular diseases. Advanced aging is associated with reduced endothelium-dependent relaxations, that is, endothelial dysfunction, in both human and animal arteries, promoting thus the initiation and development of cardiovascular diseases [3–8].

Vasorelaxation is primarily controlled by the endothelium, and both by production of endothelium-derived relaxing factors (EDRFs), as nitric oxide (NO), endothelium-derived hyperpolarizing factor (EDHF) and prostacyclin (PGI_2_), and also by endothelium-derived contracting factors (EDCFs), which reduce vasorelaxation [9]. Disbalance between EDRFs and EDCFs results in an endothelial dysfunction, which has been observed in various diseased states, including hypertension [10]. Increased EDCFs-induced vasoconstriction, mediated presumably by cyclooxygenase (COX) metabolites, participates in impairment of endothelium-dependent vasorelaxation in both genetic models of arterial hypertension and age-induced vascular changes [5, 6, 10].

Regarding vasorelaxation, NO mediates many physiological and pathophysiological functions. Age-related vascular dysfunction was shown to result mainly from reduced NO production [11, 12]. In human umbilical vein endothelial cells, aging decreased the production of NO and the activity and expression of eNOS protein [11]. Several studies indicated that aging blunted NO-dependent relaxations [8, 13–16]. All the reported studies, however, investigated vascular function of middle-aged and old (55-week- to 35-month-old) rats compared to young adult (12- to 24-week-old) rats. According to animal models and the vascular bed studied, aging-related changes can involve different mechanisms. Yet little information is available on early alterations in the NO-mediated vasorelaxation from blood vessels of juvenile and young adult rats either with or without a genetic load of hypertension.

As alterations in aging may, at least in part, depend on hemodynamic factors such as arterial pressure [14], a further aim of this study was to determine the influence of aging in young spontaneously hypertensive rats (SHR). We used SHR as this experimental model of genetic hypertension is similar to the human form of increased blood pressure (BP) and can help to better understand the mechanisms of essential hypertension in humans [17, 18]. The characteristic acceleration of BP rise in SHR (as compared with normotensive WKY rats) occurs mainly between the 3rd and 10th week of age. Over this period of age, their BP rapidly increases and continues to rise at least until the age of 20 weeks [19]. In contrast, the BP of WKY reaches adult levels by the 10th week of age [19]. High blood pressure in SHR may result from sympathetic hyperactivity and sympathetic vasoconstriction [20, 21]. In SHR, the femoral artery, a conductance medium-size artery, exhibits increased vasoconstriction to the sympathetic vasoconstrictor noradrenaline [22–24] and endothelial dysfunction in adulthood [25]. However, opposite findings were also described in the SHR femoral artery [26]. Nevertheless, pharmacological studies using the femoral artery are highly relevant for a better understanding of the pathophysiology of peripheral artery disease (PAD), whose incidence has an increasing tendency in the world population [27, 28] similar to hypertension. Many factors, including hypertension and advancing age, have been implicated in the pathogenesis of PAD [27, 29, 30]. Although the femoral arteries are not the main factor contributing to elevated peripheral resistance in hypertension, altered vascular function in early periods of life may be involved in various diseased states in old age. In addition, different mechanisms may be involved in the vascular aging in the aorta, the common femoral artery [31], and small mesenteric arteries [5] in various periods of life.

In order to determine vascular changes in early periods of life, we investigated alterations in BP, NO production, and vascular function of the isolated femoral artery of juvenile and young adult male normotensive Wistar-Kyoto (WKY) and spontaneously hypertensive rats. In addition, we determined NO-dependent and -independent components of endothelium-dependent relaxation to investigate possible compensatory mechanisms participating in the maintenance of normal vascular function in juvenescence.

## 2. Materials and Methods

### 2.1. Animals and Experimental Design

Male 7-week-old (juvenile) and 22-week-old (young adult) SHR and WKY rats were used (*n* = 8–10). All the rats used in the present study were born in our certified animal facility (Institute of Normal and Pathological Physiology SAS). The rats were housed in groups of five animals per cage, each strain separately, in an air-conditioned room at constant temperature (22–24°C) and humidity (45–60%) at a 12 : 12-h light/dark cycle (06 : 00–18 : 00 h lights on) and they were maintained on a standard pellet diet and tap water* ad libitum*. All procedures used were approved by the State Veterinary and Food Administration of the Slovak Republic.

Systolic blood pressure (SBP) and heart rate (HR) were determined noninvasively in conscious rats by the tail-cuff method at the end of the experiment as described previously [32]. Body weight (BW) was recorded at the same time. Seven- and 22-week-old rats were killed by decapitation after a brief CO_2_ anesthesia. Wet weights of the left heart ventricle (LVW) were determined for calculation of their relative weights (LVW/body weight) to evaluate the degree of cardiac hypertrophy.

### 2.2. Assessment of Vascular Reactivity of the Femoral Artery by Wire Myograph

Immediately after decapitation, the femoral artery was carefully excised and cleaned of adipose or connective tissue. The arteries were then cut into segments (1.28 ± 0.04 mm long) and mounted as ring-shaped preparations in the Mulvany-Halpern style small vessel wire myograph chamber (Dual Wire Myograph System 410A, DMT A/S, Aarhus, Denmark) to determine the vascular reactivity during isometric conditions as described elsewhere [33]. The preparations were bathed in modified physiological salt solution (PSS) oxygenated with a mixture of 95% O_2_ and 5% CO_2_ and maintained at 37°C [26, 32]. The composition of the PSS was (in mmol/L) NaCl 118.99, KCl 4.69, NaHCO_3_ 25, MgSO_4_·7H_2_O 1.17, KH_2_PO_4_ 1.18, CaCl_2_·2H_2_O 2.5, Na_2_EDTA 0.03, and glucose 5.5 (pH 7.4). The normalization procedure, the calculations for normalized inner diameter, and the experimental protocol for the femoral artery were described previously [25, 32].

Briefly, the contractile response to 125 mmol/L KCl (PSS was changed to KPSS in which NaCl was exchanged for an equimolar concentration of KCl) for 2 min was first obtained on each arterial ring followed by washings with PSS. An equilibration period of 20 min was allowed between each series of experiments. After confirming a sufficient contractile response to KPSS, experiments with noradrenaline (10^−5 ^mol/L) were started to obtain phasic and tonic contractile responses [24]. Since the arteries must be optimally precontracted to assess relaxation responses, a submaximal tone was induced with 10^−6 ^mol/L serotonin (Ser) [15, 28]. This precontraction agent was then used for all subsequent relaxation studies. When the contraction of the femoral artery to Ser reached a steady state, increasing concentrations of the vasodilator acetylcholine (ACh, 0.001 to 10 *μ*mol/L) were added in cumulative manner to perform endothelium-dependent concentration-response curves [34] followed by rinsings. To examine whether NO was involved in ACh-induced vasorelaxation of the femoral artery, N^G^-nitro-L-arginine methyl ester (L-NAME), a nonspecific NOS inhibitor, was added to the PSS at 300 *μ*mol/L and was allowed to incubate for 25 min. In the presence of L-NAME, when ACh-induced NO release could be precluded, the concentration-response curve for ACh was repeated. The drugs were then washed out (PSS, 30 min) and the nitric oxide donor sodium nitroprusside (SNP, 0.001 to 10 *μ*mol/L) was added in a cumulative fashion to assess an endothelium-independent, however NO-dependent, vascular relaxation. After the following wash-out, the femoral artery rings were stimulated again with high concentration of K^+^ (125 mmol/L) in depolarising solution to induce maximal contraction (PSS was changed to KPSS) and then left to achieve a plateau. The maximal tension reached with this depolarizing solution was set as 100% to express the active tension generated by noradrenaline [35].

NO-mediated relaxation was determined by measuring the portion of ACh-induced relaxation that was abolished by L-NAME [36, 37]. Calculations were performed by determination of the area under the curve (AUC, in arbitrary units, au) of individual dose-response curves. The NO-mediated response was then calculated as the difference between the AUC of ACh-induced relaxation in the absence and presence of L-NAME. The extent of vasorelaxation was expressed in relative values as the percentage of the initial contraction induced by Ser as well as in absolute values (mN/mm) to minimize a possible effect of different Ser-induced precontraction tone [25, 38, 39]. Vasoconstrictions were determined as the maximal tension and they were expressed as active wall tension in mN/mm. All chemicals used were purchased from Sigma-Aldrich (Germany) and Merck Chemicals (Germany), except noradrenaline hydrogenotartras (Zentiva, Czech Republic).

### 2.3. Nitric Oxide Synthase Activity

Total NO synthase (NOS) activity was measured in tissue homogenates of the aorta (200 mg/mL) by determination of [^3^H]-L-citrulline formation from [^3^H]-L-arginine (MP Biomedicals, USA, 50 Ci/mmol), as described previously [32] and expressed as pmol/min/mg of tissue proteins as determined by the Lowry method [40]. All chemicals used were purchased from Sigma-Aldrich (Germany) and Merck Chemicals (Germany).

### 2.4. Statistical Analysis

Data are presented as group mean values ± SEM of the number (*n*) of independent measurements. Results were analyzed by analysis of variance (ANOVA). Two-way ANOVA (with age and strain as independent variables) was used to compare basic biometric and cardiovascular parameters and normalized inner diameter, vascular constrictions, and nitric oxide synthase activity. In case of significant results pairwaise comparison with Bonferroni adjustment was employed. Homogeneity of variances and normality of distribution were tested by Levene's test and by Shapiro-Wilk's test, respectively. Concentration response curves were compared using two-way repeated measurements ANOVA, followed by vertical contrast with Bonferroni adjustment. To assess depression present at high concentration of ACh-cumulative concentration response curves, the maximal response, and the response at higher ACh concentration at a particular response curve was compared with Dunnett's test. Means were considered to differ significantly when *P* < 0.05.

## 3. Results

Systolic blood pressure, heart rate, body weight, LVM/BW ratio, and vascular parameters of experimental groups are shown in Table 1. SBP, heart rate, and LVW/BW were significantly increased and BW and normalized inner diameter of the femoral artery was significantly decreased in both SHR groups as compared to age-matched WKY (Table 1). LVW/BW was lower in all young adult (22-week-old) groups than in juvenile rats. A significant age-dependent increase of BW and normalized inner diameter of the femoral artery was found in 22-week-old WKY and SHR; however age-related SBP increase was present only in 22-week-old SHR compared to juvenile 7-week-old SHR.

**Figure 1:**
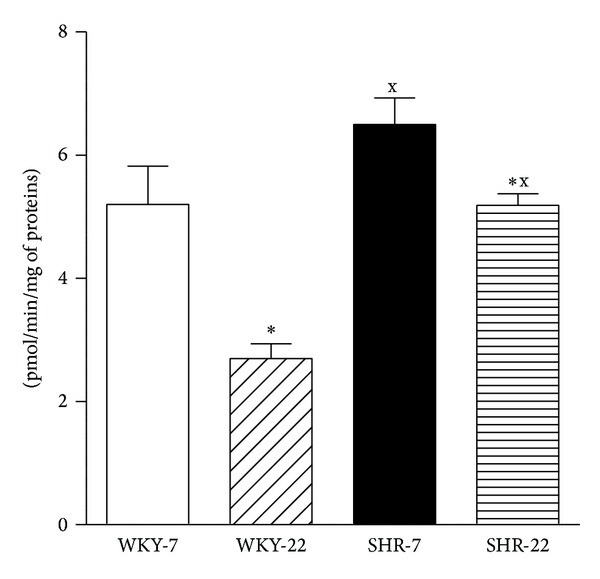
Nitric oxide synthase activity in the aorta of young adult (22-week-old) and juvenile (7-week-old) Wistar-Kyoto (WKY) and spontaneously hypertensive rats (SHR). SHR-7: 7-week-old SHR; SHR-22: 22-week-old SHR; WKY-7: 7-week-old WKY rats; WKY-22: 22-week-old WKY rats. Values represent mean ± SEM of 6–8 rats. Symbols have the following meanings: ^x^ *P* < 0.05 compared to age-matched WKY (SHR-7 versus WKY-7, SHR-22 versus WKY-22), *P < 0.05 compared to juvenile rats (WKY-22 versus WKY-7, SHR-22 versus SHR-7).

NOS activity was increased in SHR compared to age-matched WKY (Figure 1). Additionally, there were significant age-related decreases in NOS activity in the aorta of WKY and SHR (Figure 1).

**Figure 2:**
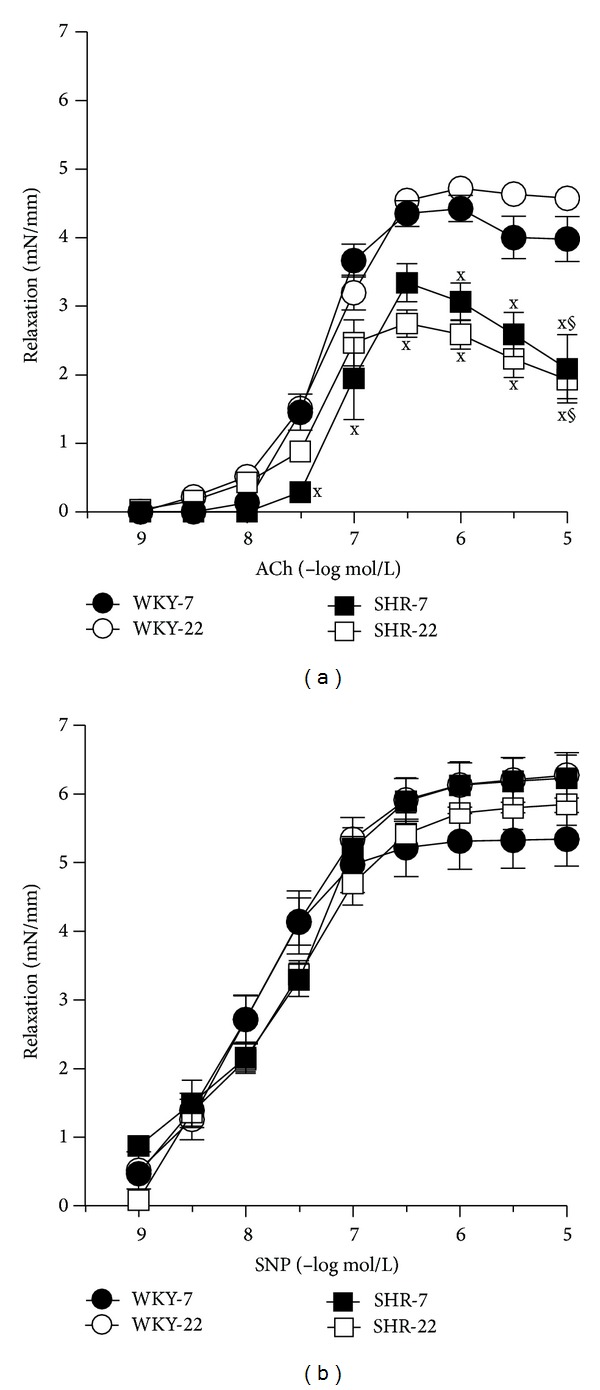
(a) Vascular responses to acetylcholine (ACh) and (b) sodium nitroprusside (SNP) in isolated femoral arteries of 7- and 22-week-old Wistar-Kyoto (WKY) and spontaneously hypertensive rats (SHR). Values represent mean ± SEM of 6–8 rats. SHR-7: 7-week-old SHR; SHR-22: 22-week-old SHR; WKY-7: 7-week-old WKY rats; WKY-22: 22-week-old WKY rats. Symbols have the following meanings: ^x^ *P* < 0.05 compared to age-matched WKY (SHR-7 versus WKY-7, SHR-22 versus WKY-22), ^§^ *P* < 0.05, compared to maximal relaxation at ACh concentrations 0.3 *μ*mol/L—this significance indicates the release of counterbalancing contracting factors in hypertensive animals.

**Figure 3:**
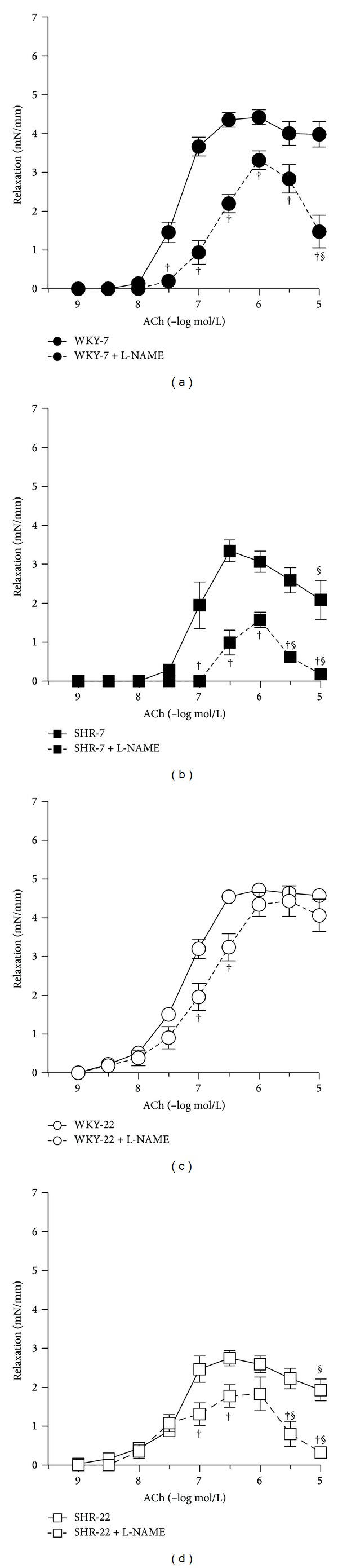
Vascular responses to acetylcholine (ACh) in isolated femoral arteries of 7- and 22-week-old Wistar-Kyoto (WKY) and spontaneously hypertensive rats (SHR) before (full lines) and after (dashed lines) incubation with the nitric oxide (NO) synthase inhibitor N^G^-nitro-L-arginine methyl ester (L-NAME, 300 *μ*mol/L). Endothelium-dependent relaxations in the absence and presence of L-NAME of (a) 7-week-old WKY, (b) 7-week-old SHR, (c) 22-week-old WKY, and (d) 22-week-old SHR. Values represent mean ± SEM of 6–8 rats. SHR-7: 7-week-old SHR; SHR-22: 22-week-old SHR; WKY-7: 7-week-old WKY rats; WKY-22: 22-week-old WKY rats. Symbols have the following meanings: ^†^ *P* < 0.05 compared to the respective value before L-NAME; ^§^ *P* < 0.05, compared to maximal relaxation at ACh concentrations 0.3 or 1 *μ*mol/L (a, c, d)—significance indicating release of counterbalancing vasocontractile factors in hypertensive animals and in L-NAME-treated femoral arteries from juvenile WKY and juvenile and young adult SHR.

ACh (1 nmol/L–10 *μ*mol/L) and SNP (1 nmol/L–10 *μ*mol/L) relaxed the femoral artery from both WKY and SHR in a concentration-dependent manner (Figure 2). Application of L-NAME abolished partially the effect of ACh, as illustrated in Figure 3. Since serotonin (1 *μ*mol/L) induced different responses in 7- compared to 22-week-old WKY arteries and also in 22-week-old WKY compared to SHR femoral arteries resulting in a different prerelaxation active tension level (Table 1), the relaxation results were quantitatively expressed as mN/mm. However, calculations for relaxation responses in percentages (the percent of relaxation calculated relative to the steady-state contraction to Ser) revealed similar differences between the experimental groups and the results correlated well with the extent of prerelaxation active tone (data not shown). ACh-induced concentration-response curves were comparable in juvenile and young adult rats (Figure 2(a)). ACh-induced vasorelaxation was lower in SHR than that in age-matched WKY (Figure 2(a)). In SHR, maximal relaxation was achieved at ACh concentration of 3·10^-7 ^mol/L and a slight contractile effect counteracted the relaxant response at higher concentrations of ACh, resulting in a significant decrease in relaxation response at 10^-5 ^mol/L ACh compared to the maximum relaxation response (Figure 2(a)). Cumulative addition of the NO donor sodium nitroprusside (SNP) produced similar relaxation responses in the femoral arteries from all experimental groups (Figure 2(b)).

**Figure 4:**
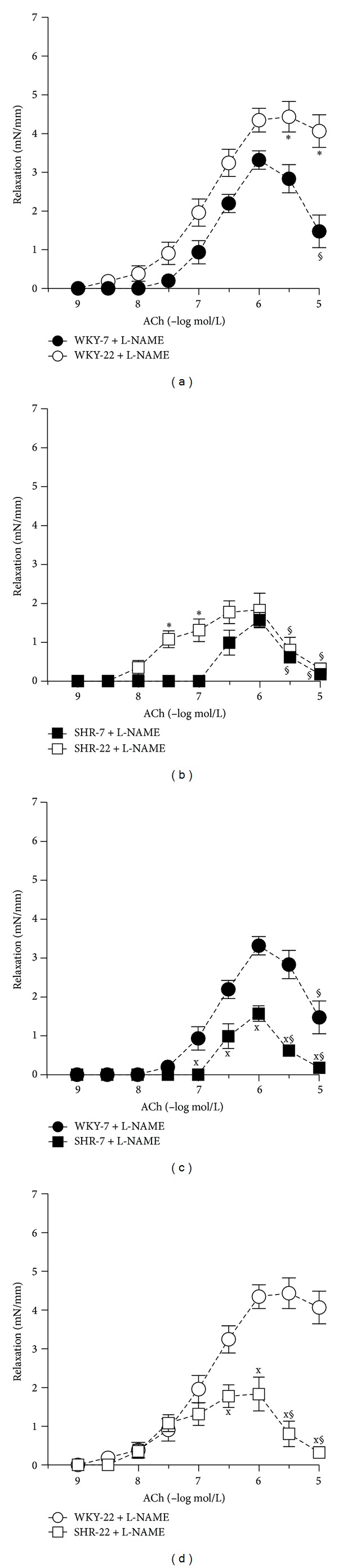
Vascular responses to acetylcholine (ACh) in isolated femoral arteries of 7- and 22-week-old Wistar-Kyoto (WKY) and spontaneously hypertensive rats (SHR) in the presence of nitric oxide (NO) synthase inhibitor N^G^-nitro-L-arginine methyl ester (L-NAME, 300 *μ*mol/L). Age-related effect on endothelium-dependent L-NAME-resistant relaxations in (a) 7- and 22-week-old WKY and (b) 7- and 22-week-old SHR. Differences between normotensive and hypertensive rats in (c) 7-week-old and (d) 22-week-old rats. Values represent mean ± SEM of 6–8 rats. SHR-7: 7-week-old SHR; SHR-22: 22-week-old SHR; WKY-7: 7-week-old WKY rats; WKY-22: 22-week-old WKY rats. Symbols have the following meanings: ^x^ *P* < 0.05 compared to age-matched WKY (SHR-7 versus WKY-7, SHR-22 versus WKY-22), *P < 0.05 compared to juvenile rats (WKY-22 versus WKY-7, SHR-22 versus SHR-7), and ^§^ *P* < 0.05 compared to maximal relaxation at ACh concentrations 0.3 or 1 *μ*mol/L (a, b, c, d)—significance indicating release of counterbalancing vasocontractile factors in hypertensive animals and in L-NAME-treated femoral arteries from juvenile WKY and juvenile and young adult SHR.

**Figure 5:**
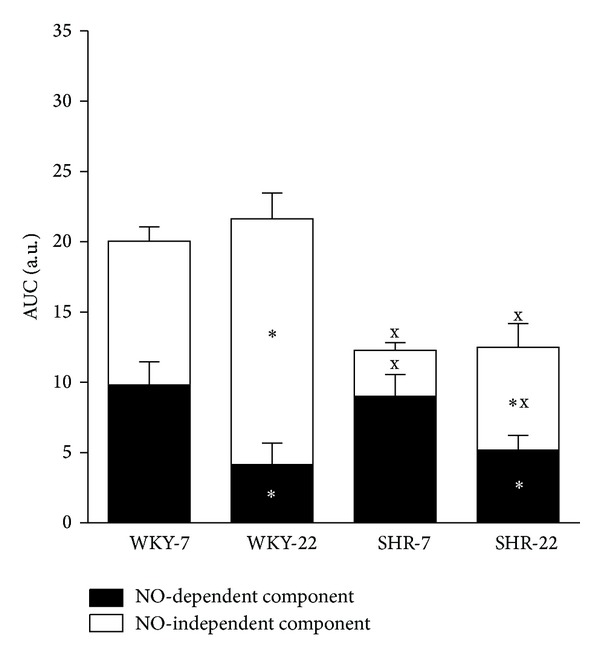
Area under the curve (AUC) of the vascular responses, based on the individual concentration-dependent relaxation curves to acetylcholine (ACh) in isolated femoral arteries of 7- and 22-week-old Wistar-Kyoto (WKY) and spontaneously hypertensive rats (SHR). Values represent mean ± SEM of 6–8 rats. SHR-7: 7-week-old SHR; SHR-22: 22-week-old SHR; WKY-7: 7-week-old WKY rats; WKY-22: 22-week-old WKY rats. Marks of significances over the entire columns represent significant differences between the total ACh-induced relaxations. Marks inside the black part indicate significances between NO-dependent component of vasorelaxation and marks inside the white bar represent the significant differences between the NO-independent components. Symbols have the following meanings: ^x^ *P* < 0.05 compared to age-matched WKY (SHR-7 versus WKY-7, SHR-22 versus WKY-22), *P < 0.05 compared to juvenile rats (WKY-22 versus WKY-7, SHR-22 versus SHR-7).

L-NAME attenuated ACh-induced vasorelaxation in all groups investigated (Figure 3). The effect of L-NAME on ACh-induced relaxation did not differ between 7-week-old and 22-week-old SHR as compared to age-matched WKY (Figures 3 and 5). However, L-NAME attenuated ACh-induced relaxation in a smaller degree in young adult 22-week-old rats in both strains as compared to juvenile 7-week-old rats (Figures 3 and 5). When the arteries were pretreated with L-NAME, the femoral arteries from 7- and 22-week-old SHR rats responded by smaller relaxation to ACh than did the age-matched normotensive WKY arteries (Figures 4(c) and 4(d)), yet the femoral arteries from young adult 22-week-old rats responded in an even higher extent than did arteries from juvenile 7-week-old rats (Figures 4(a) and 4(b)).

**Figure 6:**
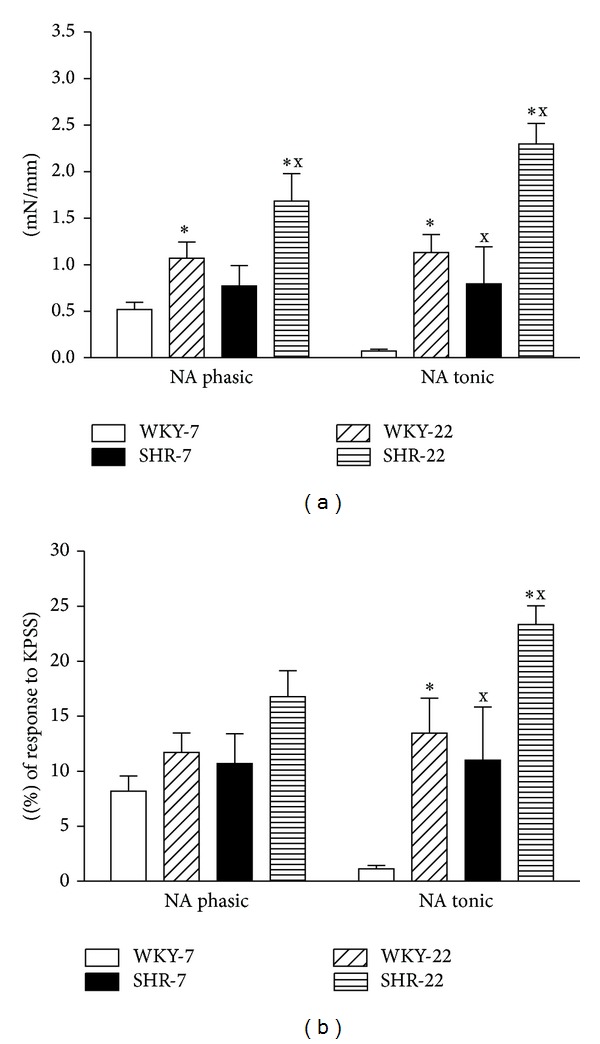
Vascular constrictions induced by noradrenaline (NA, 10 *μ*mol/L) in the femoral artery of young adult (22-week-old) and juvenile (7-week-old) Wistar-Kyoto (WKY) and spontaneously hypertensive rats (SHR). SHR-7: 7-week-old SHR; SHR-22: 22-week-old SHR; WKY-7: 7-week-old WKY rats; WKY-22: 22-week-old WKY rats. Values represent mean ± SEM of 6–8 rats. Symbols have the following meanings: ^x^ *P* < 0.05 compared to age-matched WKY (SHR-7 versus WKY-7, SHR-22 versus WKY-22), *P < 0.05 compared to juvenile rats (WKY-22 versus WKY-7, SHR-22 versus SHR-7).

NA-induced responses were biphasic: a transient contraction (early response, phasic contraction), which occurred within the first 10–15 sec and returned nearly to baseline was followed by sustained contraction (delayed response, tonic contraction), which reached steady maximum levels at 5 to 20 min. The tonic response of the 7- and 22-week-old SHR femoral artery to noradrenaline was greater than that in the age-matched WKY (Figure 6(a)). Phasic response was augmented in the 22-week-old SHR compared to the age-matched WKY. Aging significantly potentiated the contractions induced by NA in the femoral artery of both SHR and WKY. Unlike the NA-induced contraction, the KCl-induced contraction of the femoral artery seems to be unaffected in hypertensive rats, though an age-related increase was observed both in WKY and SHR (Table 1). Consequently, the ratio between the two contractile agents (NA/KCl) was still higher in the femoral artery of SHR as compared to that of WKY (Figure 6(b)). However only the tonic contraction induced by NA determined in relative values (calculated as percentage of maximal response induced by KPSS) was significantly greater in SHR than in WKY rats.

## 4. Discussion

The possible role of aging affecting vascular responses was studied in relation to experimental hypertension. In this study, we investigated age-related alterations in acetylcholine-induced relaxation of the femoral artery, its L-NAME-sensitive and -resistant components, and total nitric oxide synthase activity in the aortas of juvenile and young adult WKY and SHR. The present study showed that (1) SBP was augmented in 7- and 22-week-old SHR compared to age-matched WKY and age-related augmentation of SBP was seen in SHR but not in WKY; (2) ACh-induced vasorelaxation in the femoral artery in 7-week-old rats did not differ from that in 22-week-old rats in either strain investigated; (3) ACh-induced relaxation in 7- and 22-week-old SHR was attenuated compared with that in age-matched WKY and this endothelial dysfunction originated from reduced NO-independent mechanisms and/or elevated release of EDCF, yet not from NO deficiency; (4) ACh-induced relaxation mediated by NO and NOS activity were attenuated in young adult (22-week-old) WKY and SHR compared with that in juvenile (7-week-old) rats; (5) NA-induced vasoconstriction was augmented in 7- and 22-week-old SHR compared to age-matched WKY; and finally, (6) aging augmented the responses to NA in both WKY and SHR.

In this study we determined NO synthase activity in the aorta and the L-NAME-sensitive component of ACh-induced relaxation in the femoral artery as measures of NO production and bioavailability in the vasculature. Although we could not measure NOS activity in the femoral arteries (due to insufficient amount of tissue for this method), it is assumed that, despite anatomic heterogeneity of the aorta and femoral artery [31], changes in NOS activity in the aorta correspond to those in NO-dependent relaxation of the femoral artery, as this association was shown previously in rats with blunted NO production [37]. Additionally, it is well known that acetylcholine-induced endothelium-dependent relaxation involves besides NO also other endothelium-derived relaxing factors. We therefore investigated vascular reactivity also in the presence of the nonspecific NOS inhibitor L-NAME, in order to assess NO-dependent (i.e., L-NAME-sensitive) and NO-independent (i.e., L-NAME-resistant) relaxation. Thus changes in NO production along with changes in the NO-dependent component of ACh-induced relaxation are clear indicators of NO bioavailability in the given artery.

Using this experimental approach, we observed that aging (in a relatively early period of life) reduced the total activity of NOS in the aorta and NO-dependent vasorelaxation of the femoral artery in both WKY and SHR. These alterations were however not associated with quantitatively blunted endothelium-dependent relaxations, as determined by the ACh test.

Data from the literature suggest that aging alters vascular function. Several studies have indicated that endothelium-dependent relaxations might decline with age. Aging was found to blunt NO-dependent relaxations in the mesenteric artery of male Wistar rats [8], in the aorta of healthy normotensive male and female rats [13, 14], in the common carotid artery of normotensive WKY and spontaneously hypertensive male rats [15], and in the coronary arterioles from Sprague-Dawley male rats [16], but it did not decrease relaxation in the pulmonary artery of normotensive female rats [14].

In this study, despite unchanged total ACh-induced relaxation, we observed significant qualitative age-related changes in the mechanism of relaxation by acute preincubation with L-NAME. NO-dependent components of the ACh-induced relaxation were reduced in young adult rats in association with reduced NOS activity in both strains investigated. Although we did not measure the activity of individual NOS isoforms, there are studies indicating the participation of eNOS and iNOS in age-related alterations [11]. In contrast, during a period of life comparable to that investigated in our study (4 weeks versus 14–17 weeks), reduction of eNOS activity was not observed in the aortas of WKY and SHR [41]. Chou et al. [41] found reduced activity of eNOS only in the aortas of WKY and that in much older rats (63-week-old). In addition, the same study showed that the basal activity and protein expression of iNOS were detected only in elderly (63-week-old) SHR and WKY and in adult (14-to-17-week old) SHR yet not in adult WKY, suggesting that the abnormal expression of iNOS is associated with hypertension. This abnormal iNOS expression might be implicated also in our study concerning the finding that SHR exhibited a significantly increased NOS activity than did age-matched WKY rats. Thus the observed increased total NOS activity in the aortas from SHR as compared to WKY at both ages studied may be related to the abnormal expression of iNOS or to a compensatory mechanism by eNOS activated in hypertension [42, 43]. However, elevated NOS expression need not be always associated with improved enzyme activity. Indeed, in the aortas from aged male Wistar rats, the expression of eNOS isoform was enhanced; however its activity was markedly reduced [44]. Reduced activity of NOS was also found in this study and may account for age-related decrease of NO-mediated vasorelaxation, independently of NO breakdown by reactive oxygen species [5].

Interestingly, we observed a significant age-related increase in the L-NAME-resistant (i.e., NO-independent) component of ACh-induced relaxation in WKY and SHR. This result is in contradistinction to previous reports which showed a rather decreased EDHF-mediated relaxation in vascular aging [8]. The disparity with our results may be related to differences in age and vascular bed of the animals studied. Although it is assumed that elevation of the NO-independent component of vasorelaxation in young adult rats (versus juvenile) was elicited by compensatory release of EDHF(s) and/or vasodilatory prostanoids, the effect of other endothelium derived factors cannot be ruled out.

Our findings also indicate that other than NO-mediated mechanisms can effectively be activated to compensate the loss of NO bioavailability in early vascular aging. This explanation has been suggested in the study of Sofola et al. [45] who indicated that EDHF might compensate the loss of NO and preserve the endothelium-dependent relaxation in the mesenteric arteries of hypertensive rats in which hypertension was induced by a high-salt diet. Moreover, compensatory EDHF production has been suggested to occur in normotensive rats after acute NOS inhibition [46]. In addition, the compensatory release of vasodilatory prostanoids may be involved [5, 47].

Nevertheless, our study showed that NO-independent endothelial dysfunction was present in SHR even at the age of 7 weeks. Similarly did Mori et al. [36] show that the EDHF-mediated responses were attenuated before the loss of NO-mediated dilatation in the femoral resistance arteries of SHR compared to WKY. Our results also indicated that the effect of L-NAME on vasorelaxation did not differ in WKY and age-matched SHR; thus there were no significant differences in NO-mediated responses. Similarly, in resistance femoral arteries, there were no differences in NO-mediated vasodilatation in 5- and 15-week-old SHR compared to WKY [36]. We also assume that the decrease of NO-independent relaxation of the SHR femoral artery may be related to a reduced EDHF-dependent component because PGI_2_ inhibition by indomethacin failed to show any effects on ACh-induced relaxation of hindlimb arteries of SHR and WKY at various periods of life [36]. Yet as there is a complex cross-talk among individual EDRFs [10], the contribution of individual EDRFs to alterations of NO-independent components of ACh-induced relaxation needs to be evaluated in specifically designed studies.

Regarding vasoconstriction, elevated responses to serotonin were observed in all groups investigated after NOS inhibition. As the active tone level was decreased (not increased) in young adult SHR and comparable in the arteries from juvenile rats, it is not assumed that the level of precontraction tone could account for the reduced endothelial relaxation in SHR as compared to age-matched WKY. We found an increase in NA-induced vasoconstriction in hypertensive rats compared to normotensive age-matched controls and aging augmented further the noradrenaline-induced contractions of femoral arteries in SHR. This finding is in contrast to the report of Konishi and Su [26] showing unaffected responses to NA in 15-16-week-old SHR femoral arteries. However, other investigators [22, 23] reported similar augmentations of NA responses in the femoral artery of 16–20-week-old male SHR compared to age-matched normotensive rats. A similar augmentation of NA-induced responses was found in the aorta of male SHR [48] and the femoral artery of adult female SHR [49]. Since KCl acts directly on smooth muscle cells [25], the augmented NA response observed in SHR, in relation to KCl, indicates alterations in NA receptors and/or signaling, alterations of Ca^2+^ handling and sensitivity [22, 50–52], and structural remodeling of the arteries. Furthermore, EDCF contribution to NA-induced contraction of femoral arteries was reported to be similarly enhanced in adult (7-month-old) SHR and aged (14-month-old) normotensive WKY rats [39]. In addition, endothelium-dependent contractions elicited by high concentrations of acetylcholine were described both in hypertensive and in aged normotensive rats [53–55]. Cyclooxygenase-dependent EDCF production was found to be characteristic of aging [5, 56]. Our findings also support the role of EDCF(s) in hypertension-related endothelial dysfunction in SHR (Figures 3(b) and 3(d)). Moreover, augmented EDCF(s)-induced decline in relaxation responses after L-NAME-pretreatment in both SHR groups and juvenile WKY suggests neutralization of EDCF(s) action by NO. This mechanism was reported previously by Auch-Schwelk et al. [57].

It has been widely accepted that aging and hypertension do not affect sensitivity of vascular smooth muscle to NO (e.g., relaxation induced by endothelium-independent vascular smooth muscle relaxants such as sodium nitroprusside) and yet some conflicting findings were reported [15, 58, 59]. In our experiments, SNP-induced concentration-dependent relaxation did not differ between the groups, indicating that a decrease in the NO-mediated ACh-induced relaxations was due to specific impairment of the endothelium-dependent mechanism rather than to changes in vascular smooth muscle cells. Several studies have suggested that reduced NO bioavailability during aging can be related to increased production of reactive oxygen species. Indeed, aging was associated with blunted endothelium-dependent relaxations and excessive vascular formation of reactive oxygen species in Wistar, SHR, and WKY rats [8, 60]. It should however be noted that the oxidative stress theory of aging has to be further elucidated due to significant animal-strain-related differences in the effect of reactive oxygen species [61, 62].

It is well known that elevated vascular resistance along with increased stiffness of conduit arteries participate in aging and in hypertension development [2, 63]. We found that SBP increased significantly with aging in SHR, which was not observed in WKY. Thus chronic exposure of the femoral artery to higher pressure may not be the primary cause of age-related decrease of NO synthase inhibitor sensitive response to ACh. Moreover, our results suggest that age-related elevation of blood pressure between the 7th and 22nd week of life in SHR does not result from worsened endothelium-dependent relaxation but rather from elevated sympathetic vasoconstriction.

An elevated sympathetic tone in SHR was shown previously [19, 20] and our observation of increased heart rate in SHR at both ages studied is in agreement with these studies. Moreover, it has been reported that the hyperactivity of the sympathetic nervous system could induce structural and functional alterations of the heart and blood vessels in SHR [20, 64–66]. In addition, age-related decrease in the NO-dependent relaxation in the femoral artery along with its structural remodeling may be implicated in the pathogenesis of peripheral artery disease. Further studies are however needed to evaluate the underlying factors and the exact mechanism of age-related decrease in NO-dependent vasorelaxation in the femoral artery and their involvement in the pathogenesis of peripheral artery disease and hypertension.

## 5. Conclusions

In our study aging between the 7th and 22nd week of life was associated with decreased vascular NO production and NO-mediated vasorelaxation. However reduction in NO bioavailability did not result in endothelial dysfunction as the reduction of NO-dependent component of relaxation was fully compensated by accentuation of NO-independent relaxation in both WKY and SHR. The results suggest that NO-independent mechanisms can act as a salvage system to maintain endothelial function in situations associated with decreased NO bioavailability, at least in early periods of life. The exact role of NO in aging and hypertension remains however still open.

## Figures and Tables

**Table 1:** Age-related effect on basic biometric and vascular parameters of the femoral artery of juvenile and young adult Wistar-Kyoto (WKY) and spontaneously hypertensive rats (SHR).

	WKY		SHR	
	7 weeks	22 weeks	7 weeks	22 weeks
BW (g)	168 ± 8	416 ± 12*	135 ± 4^x^	382 ± 8^∗x^
BP (mmHg)	114 ± 2	106 ± 2	159 ± 4^x^	191 ± 3^∗x^
HR (bpm)	419 ± 13	405 ± 14	545 ± 10^x^	521 ± 19^x^
LVW/BW (mg/g)	1.99 ± 0.03	1.44 ± 0.03*	2.35 ± 0.06^x^	2.18 ± 0.05^∗x^
ND (*μ*m)	649 ± 17	769 ± 12*	556 ± 20^x^	690 ± 13^∗x^
Ser (mN/mm)	5.63 ± 0.25	7.41 ± 0.15*	5.90 ± 0.40	6.05 ± 0.50^x^
Ser_L-NAME_ (mN/mm)	7.03 ± 0.45^†^	8.94 ± 0.31^∗†^	7.44 ± 0.38^†^	7.80 ± 0.47^†^
*E*_max ACh_ (mN/mm)	4.50 ± 0.21	4.80 ± 0.15	3.40 ± 0.25^x^	2.83 ± 0.19^x^
*E*_max L-NAME_ (mN/mm)	3.38 ± 0.25^†^	4.54 ± 0.36*	1.57 ± 0.20^x†^	1.99 ± 0.40^x†^
*E*_max SNP_ (%)	95.3 ± 0.9	93.9 ± 1.0	98.6 ± 0.8	94.0 ± 0.8
KPSS (mN/mm)	6.54 ± 0.61	9.17 ± 0.90*	7.42 ± 0.63	9.78 ± 0.48*

ACh: acetylcholine; BW: body weight; BP: blood pressure; *E*_max ACh_: maximal acetylcholine-induced relaxation based on individual concentration-response curves; *E*_max L-NAME_: maximal acetylcholine-induced relaxation after L-NAME based on individual concentration-response curves; *E*_max SNP_: maximal sodium nitroprusside-induced relaxation based on individual concentration-response curves; HR: heart rate; KPSS: high-potassium physiological salt solution; L-NAME: N^G^-nitro-L-arginine methyl ester; LVW: left heart ventricle weight; ND: normalized diameter of the femoral artery at 13.3 kPa calculated within the normalization procedure; Ser: vascular constriction induced by serotonin (1 *μ*mol/L); Ser_L-NAME_: vascular constriction induced by serotonin (1 *μ*mol/L) after L-NAME; SNP: sodium nitroprusside. Results are mean ± S.E.M. of 6–10 rats. ^x^ *P* < 0.05 compared to age-matched WKY (SHR-7 versus WKY-7, SHR-22 versus WKY-22), *P < 0.05 compared to juvenile rats (WKY-22 versus WKY-7, SHR-22 versus SHR-7), and ^†^ *P* < 0.05 compared to the respective value before L-NAME.
